# Real-Time Identification and Quantification of Per- and Polyfluoroalkyl Substances Using High-Resolution Time-of-Flight Chemical Ionization Mass Spectrometry with Positive Reagent Ions

**DOI:** 10.1021/acs.analchem.5c02489

**Published:** 2025-12-25

**Authors:** Sahir Gagan, Miska Olin, Alana J. Dodero, Siddharth Gopalakrishnan, Sining Niu, Michael J. Davern, Barbara J. Turpin, Jason D. Surratt, Yue Zhang

**Affiliations:** † Department of Atmospheric Sciences, 14736 Texas A&M University, College Station, Texas 77843, United States; ‡ Department of Chemistry, College of Arts and Sciences, 2331 University of North Carolina at Chapel Hill, Chapel Hill, North Carolina 27514, United States; § Department of Environmental Sciences and Engineering, Gillings School of Global Public Health, University of North Carolina at Chapel Hill, Chapel Hill, North Carolina 27599, United States

## Abstract

Per- and polyfluoroalkyl substances (PFAS) are emerging pollutants of concern, primarily due to their terminal degradation products, which exhibit environmental persistence and mobility. Several groups of PFAS, including hydrofluoroolefins (HFOs), perfluoro olefins (PFOs), perfluoro vinyl ethers (PVEs), and hydrofluoroalkanes (HF-alkanes), are volatile and reside predominantly in the gas phase. PFAS such as HFOs, PFOs, and PVEs are considered reactive and may generate short-chain degradation products that persist in the environment. Despite the importance of these gaseous PFAS, there is a lack of analytical techniques capable of providing high-resolution temporal measurements of potential precursors to terminal degradation products. This study presents the first real-time method for detecting and quantifying atmospheric HFOs, PFOs, PVEs, and HF-alkanes using a high-resolution chemical ionization mass spectrometer (HR-CIMS). Using NO^+^ mixed with O_2_^+^ (NO^+^/O_2_^+^), and O_2_^+^ as reagent ions, the CIMS was able to identify and quantify PFAS via fluoride abstraction (M – F)^+^, hydride abstraction (M – H)^+^, or charge transfer (M^+^) mechanisms. The method achieves 10-s limits of detection (LOD) ranging from 2 to 40 ppt, enabling online monitoring in ambient air, especially near emission sources or in indoor environments. The use of NO^+^/O_2_^+^ and O_2_^+^ reagent ions with HR-CIMS provides a novel and sensitive approach for real-time detection of PFAS via positive reagent ion modes, especially for emerging gas-phase PFAS that currently lack suitable online measurement techniques to better constrain their atmospheric emissions and concentrations.

## Graphical abstract

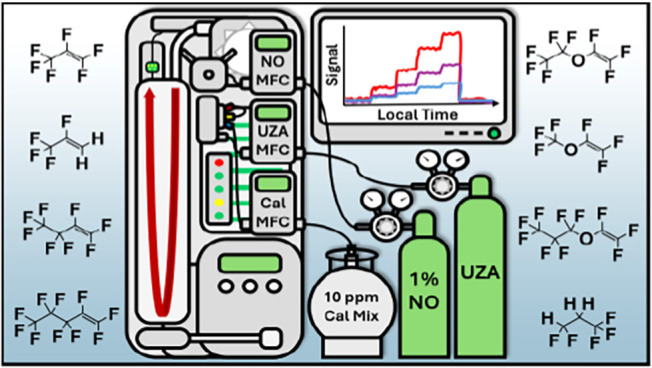

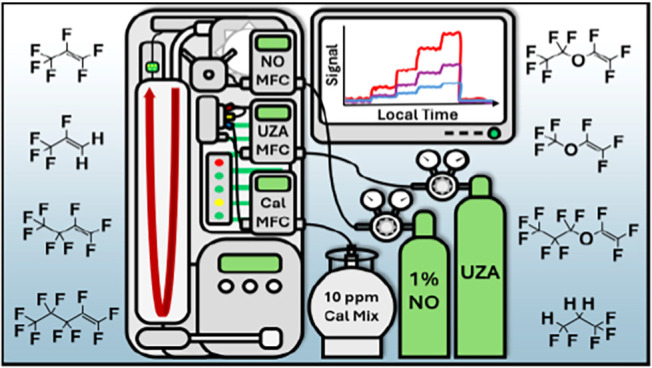

## Introduction

Per- and polyfluoroalkyl substances (PFAS) are a class of anthropogenic chemicals^1,2^ used in various consumer and industrial applications (e.g., as fire extinguishers,^3–5^ paints,^6^ water-resistant textiles^4,7^ ). Fluorinated gases are well-recognized greenhouse gases,^8,9^ and emissions of compounds such as HFO-1234yf, widely used as a refrigerant in automobile air conditioners and as a heat transfer fluid, are reported to be on the rise.^10,11^ Additionally, terminal degradation products of many PFAS, most commonly perfluoroalkylcarboxylic acids (PFCAs), are known to persist,^12^ and their widespread presence^13^ negatively influences human health^14–17^ and the environment.^18^

To assess secondary emissions of PFCAs, it is crucial to examine the chemical transformation of their precursor compounds, whose emissions may be increasing, as existing policies have primarily targeted direct PFCA emissions.^19,20^ For instance, a recent study reported that the installation of a thermal oxidizer at a manufacturing facility effectively reduced emissions of PFAS, including GenX.^21^ However, the same study also found that carbon bed adsorption systems within the facility were unable to adequately capture volatile PFAS with boiling points below 100 °C. While such regulatory and technological measures have mitigated long-chain PFAS emissions, they may have inadvertently shifted releases toward shorter-chain precursors, such as those investigated in the present study. Short-chain gas phase PFAS (C_3_–C_6_) may act as a precursor to short-chain persistent PFCAs through atmospheric oxidation,^10,22^ which is still an environmental and health concern. A recent study has shown the presence of persistent short-chain PFCA (carbon ≤ 3) in the remote location of the high Arctic of Canada.^23^ Another study conducted real-time measurements of C_2_–C_6_ PFCAs and showed evidence of secondary formations of these short-chain PFCAs.^11^ In addition, the photodegradation of refrigerant HFO-1234yf^10,22^ is known to form trifluoroacetic acid (TFA). TFA has been correlated with elevated fasting glucose levels and glycated hemoglobin (HbA1c), which is associated with an increased risk of diabetes and related complications.^15^ These adverse environmental and health effects highlight the importance of quantifying the concentrations and emissions of atmospheric gas-phase PFAS.

Offline techniques are commonly used to sample airborne PFAS. Time-integrated samples with various substrates (e.g., PUF-XAD cartridges), subsequently interfaced with gas chromatography (GC) or liquid chromatography mass spectrometry techniques,^24–29^ are often used to measure PFAS. Previously, a method has been developed to measure total gaseous fluorine via platinum-catalyzed thermolysis, and ambient gaseous fluorine has been measured via impinger-based collection.^30^ While integrated sampling and mass spectral analysis can provide high confidence in compound identification, offline sampling typically does not provide high enough temporal resolution to pinpoint the influence of particular sources and activities,^29^ and it is prone to sampling, storage, and handling artifacts that increase measurement uncertainties.^24,29^ To address these shortcomings, it is critical to develop alternative techniques that can provide real-time quantification of PFAS. Recently, high-resolution time-of-flight chemical ionization mass spectrometery (HR-ToF-CIMS) methods equiped with either iodide or acetate as a reagent ion have been used to characterize and quantify various fluorotelomer alcohols (FTOHs) and PFCAs.^3,11,31^ Riedel et al.^3^ reported the first gas-phase detection of fluorotelomer alcohols (FTOHs) and PFCAs, and performed calibrations using I-HR-ToF-CIMS that were then used to quantify FTOHs emitted from commercially available fluoro-surfactants and aqueous film-forming foam (AFFF) concentrate.^3^ Davern et al.^31^ reported improved sensitivities for FTOHs and demonstrated the ability to measure concentrations relevant to the indoor environment when they measured gas-phase concentrations of these PFAS from microwave popcorn and rain jackets. I-HR-ToF-CIMS has also been used to characterize perfluoroalkyl sulfonic acids (PFSA) and perfluoroalkyl phosphoric acid diesters (diPAP).^32^ Young et al.^11^ recently developed a method using acetate-CIMS to constrain the organic fluorine burden and detect short-chain PFCAs to parts per quadrillion levels (ppq). However, I-HR-ToF-CIMS and acetate-CIMS are not sensitive to PFAS with certain functional groups, including per-/polyfluorinated olefins (PFOs), hydrofluoroalkanes (HF-alkanes), and perfluorinated vinyl ethers (PVEs).^3,11,31,33^ For instance, a recent study by Mattila and Offenberg^33^ reported the TFA emission in ambient air in New Jersey using I-HR-ToF-CIMS, where HFO-1234yf was hypothesized as a potential source but was not detectable by the I-HR-ToF-CIMS. D’Ambro et al. modeled significant PFO and PVE compound emissions near a PFAS manufacturing source, but lacked real-time atmospheric measurement techniques to validate predictions.^34^ Hence, developing a real-time measurement method to characterize and quantify such PFAS categories is imperative to assess their sources, transformations, fate, and implications to human health.

The application of positive ion chemistry to selected non-PFAS VOCs has been previously studied using selected-ion flow tube mass spectrometry (SIFT-MS).^35,36^ NO^+^ and O_2_^+^ were used as new reagent ions to address some of the limitations of H_3_O^+^ for the purpose of improving identifications of carbonyl isomers and reducing fragmentation of large hydrocarbons (C8 and larger). Although the NO^+^ reagent ion addresses the limitation of H_3_O^+^, it inherently generates the O_2_^+^ reagent ion simultaneously.^35–37^ By adjusting the applied voltages, the relative ratio of NO^+^ and O_2_^+^ can be tuned, however, it is not possible to eliminate O_2_^+^.^37,38^ In recent years, the mixed ionization NO^+^ and O_2_^+^ CIMS technique has been widely explored for the characterization of alkanes,^38,39^ alkenes,^37,40^ cycloalkanes,^41^ and other VOCs.^37^ Previous studies have used mixed ionization of NO^+^ and O_2_^+^ reagent ions as well as optimized NO^+^ reagent ions to characterize evaporative emissions from gasoline-powered cars,^42^ characterize the isomers of acetone and propanal,^37^ and *n*-alkanes in complex oil mixtures.^43^ However, such innovative ionizations have not been widely studied, especially with fluorine-containing species such as PFAS.

The current study addresses the key knowledge gap in time-resolved CIMS by providing the first real-time characterization and quantification of three previously unreported classes of PFAS (i.e., PFOs, HF-alkanes, and PVEs) using NO^+^ mixed with O_2_^+^ (herein referred to as NO^+^/O_2_^+^), O_2_^+^, and H_3_O^+^ as reagent ions. Compared with the H_3_O^+^ reagent ion mode, NO^+^/O_2_^+^ and O_2_^+^ reagent ions are highly sensitive toward these PFAS classes of compounds and can reach detection limits as low as ppt levels, highlighting the potential for innovative applications of ambient gas-phase PFAS measurements near or away from point sources using NO^+^/O_2_^+^ or O_2_^+^ reagent ions.

## Experimental Section

### Materials

Pure standards for the gas-phase PFAS, including 2,3,3,3-tetrafluoropropene (HFO-1234yf, 99%), 1,1,1,2,3-pentafluoropropane (PFP, 99%), perfluoromethyl vinyl ether (PMVE, 99%), perfluoroethyl vinyl ether (PEVE, 99%), perfluoropropyl vinyl ether (PPVE, 99%), hexafluoropropylene (HFP, 99%), perfluoro-2-butene (PFB, 99%), and perfluoro pentene (PFPe, 95%), were purchased from Synquest Laboratories. A (10.0 ± 0.1) ppm (v/v ratio) reference mixture of eight PFAS balanced by N_2_ (Nutech Instruments, Plano, TX, USA), prepared from pure standards (Supporting Information Section S1), was used to calibrate the Vocus 2R HR-ToF-CIMS (Aerodyne Research Inc./TOFWERK AG), for PFAS analysis. The chemical structure of each PFAS standard is shown in Figure S1. A 14-component volatile organic compound (VOC) reference mixture (Apel-Riemer Environmental, Inc. Miami, FL, USA) was used to tune the voltage settings of the Vocus 2R HR-ToF-CIMS with NO^+^/O_2_^+^ reagent ion, optimizing reagent ion distribution, sensitivity, and mass resolution. A list of compounds in the VOC reference mixture is provided in Table S1. A commercially available pure HFO-1234yf refrigerant gas cartridge was also used to simulate ambient measurement of HFO-1234yf.

### Instrumentation

We used Vocus 2R HR-ToF-CIMS (CIMS, ∼9400 *m*/Δ*m*), with NO^+^/O_2_^+^, O_2_^+^, and H_3_O^+^ reagent ions. Details of the working principle of CIMS have been previously reported.^44^ The reagent gas for NO^+^/O_2_^+^ was either ultrapure zero air (UZA) or NO 1% (v/v ratio of NO, balanced by nitrogen), and O_2_^+^ was generated using ultrahigh purity (UHP) O_2_ gas, with a flow rate of 5 sccm through the ion source. The ion source consists of two conical surfaces that produce plasma with a voltage of ∼370 V (when using UZA) or ∼405 V (when using NO 1%) to charge the reagent gas, with the charge current regulated at 2.0 mA. The reagent ion gas then entered the focusing ion molecule reactor (FIMR), operated at ∼2.2 mbar. Then the big-segmented quadruple (BSQ, ∼7 mbar) focused the ion beam before it entered the primary beam region. Finally, the ion beam entered the ToF-MS (1 × 10^–6^ mbar)^44^ and was then detected by microchannel plates (MCP).

## Result and Discussion

### Reagent Ions for CIMS

#### H_3_O^+^ Reagent Ion

The CIMS has been used previously with deionized water to generate H_3_O^+^ reagent ions. The working principle and information regarding the use of H_3_O^+^ to detect VOCs is described previously.^44–46^ Briefly, H_3_O^+^ interacts with the parent molecule to form a cluster ion by adding a proton to the parent molecule (R1).

R1 $$M+H_{3}O^{+}\to {\left[MH\right]}^{+}$$

H_3_O^+^ is often considered a semisoft ionization and may lead to fragmentation for molecules with functional groups, such as olefins and alkanes, making detection more challenging and reducing overall sensitivity.

#### NO^+^ Reagent Ion

NO^+^ can detect compounds via three pathways: via hydride abstraction (HA, R2), a charge transfer (CT, R3), and NO^+^ cluster formation (CF, R4).

R2 $$M+{NO}^{+}\overset{HA}{\to }{\left[M-H\right]}^{+}+\left(NO+H^{-}\right)$$

R3 $$M+{NO}^{+}\overset{CT}{\to }M^{+}+NO$$

R4 $$M+{NO}^{+}\overset{CF}{\to }{\left[MNO\right]}^{+}$$

For NO^+^/O_2_^+^, the sensitivity of a desired PFAS analyte was optimized by tuning the FIMR front voltage and BSQ voltage.^44^ The voltage scans for the front FIMR were varied from 350 to 630 V, while keeping the BSQ voltage constant at 150 V. The *E*/*N*, where *E* is the electric field strength in the FIMR and *N* is the concentration of neutral molecules, changes with the change in the FIMR front voltage. *E*/*N* determines the amount of reagent ions that pass through the FIMR and the reagent ion cluster formation with the target molecules, as explained in detail in Supporting Information Section S2 and eq S6.^44^ Figure 1a,b show the behavior of select reagents and interference ions in units of counts per second (cps) against *E*/*N*. To optimize the *E*/*N* for better sensitivity for targeted PFAS, an 8-component PFAS reference mixture (10.0 ppm ± 0.1, v/v ratio) diluted to concentration of 19.9 ppb was introduced into the CIMS. With increasing *E*/*N*, the signal of the target PFAS also increases (Figure 2). It should be noted that at lower *E*/*N* values, UZA generated a higher ratio of NO^+^ to O_2_^+^ as compared to NO 1% as the reagent ion source (Figure 1a,b). But higher sensitivities are observed for target PFAS in both cases at *E*/*N* ∼ 135, where the signal intensity for NO^+^ and O_2_^+^ is similar. Since the objective of the current study is to maximize the sensitivity for targeted PFAS, the method was optimized as mixed ionization of NO^+^ and O_2_^+^. The increase in sensitivity is likely due to the concurrent increase in both NO^+^ and O_2_^+^ with increasing *E*/*N*, and an approximately equal ratio of both the reagent ions at *E*/*N* ∼ 135 could result in higher sensitivities observed for targeted PFAS. Based on such results, the instrument was tuned and further calibrated at higher *E*/*N* (∼135 Td) to achieve higher sensitivity. A voltage scan was also performed for O_2_^+^, where O_2_^+^ signal was found to be dominant across all *E*/*N* values (Figure 1 c). Higher *E*/*N* is not always recommended due to the potential for more fragmentation at higher values. As reported in previous studies,^37,43^ once the reagent ion concentration was optimized, a series of calibrations were carried out to optimize the BSQ voltage using PFAS standards. In the CIMS, the BSQ both filters out low-molecular-weight ions and focuses the ion beam toward the center.^44^ These combined effects introduce molecular weight dependence in the observed ion distribution to those generated in the reactor.^44^ Specifically, low-mass ions are transmitted through the BSQ with lower efficiency than the higher-mass ions.^44^ As a result, the presence of BSQ limits the ability to normalize ion signals against primary ions such as NO^+^ (*m*/*z* 30) and O_2_^+^ (*m*/*z* 32), leading to potential differences in the primary ion intensities compared with previous research.^37^

**Figure 1:**
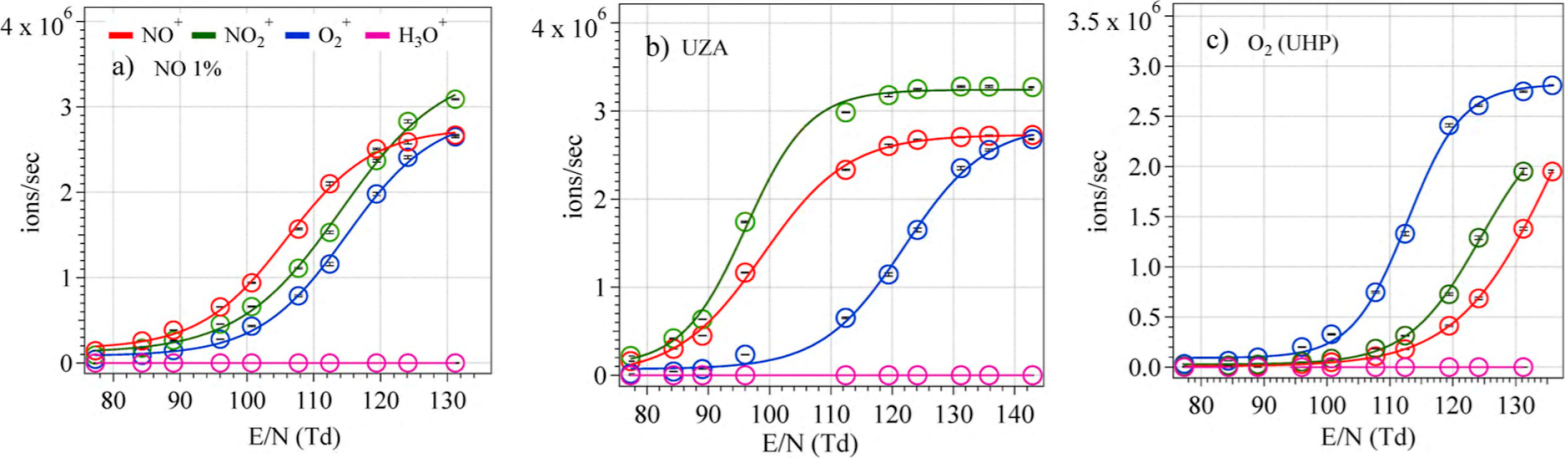
Signal intensity (ions/sec) for NO^+^, O_2_^+^, NO_2_^+^, and H_3_O^+^ as a function of *E*/*N* with NO^+^ and O_2_^+^ as reagent ions. (a) NO 1% (balanced by N_2_) is used as a source to generate the reagent ion, (b) Zero air (UZA) is used as a source to generate the reagent ion. (c) O_2_ (UHP) is used to generate the O_2_^+^ reagent ion. The error bar represents standard deviation over the signal (ions/sec), but is small and falls within the marker.

**Figure 2:**
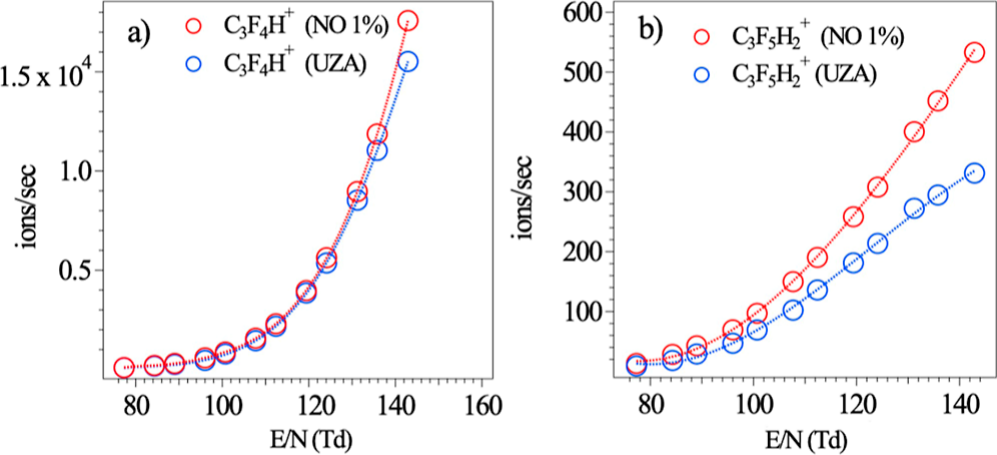
Detected signals for (a) 2,3,3,3-tetrafluoropropene (C_3_F_4_H^+^) and (b) 1,1,1,2,3 pentafluoropropane (C_3_F_5_H_2_^+^) as a function of *E*/*N* with NO^+^/O_2_^+^ as a reagent ion.

The BSQ voltage varied from 150 to 380 V, while the front voltage for FIMR was kept at 600 V. This led to an increased sensitivity of the PFAS species by a factor of 3 compared with the BSQ 150 V and FIMR 630 V (Figure S2). A higher BSQ voltage of 380 V was used to optimize the method to achieve better sensitivity. The optimized voltage settings used two different NO^+^/O_2_^+^ reagent ion sources, UZA and NO 1%, to calibrate the PFAS and compare the sensitivity between the sources. Figure 2 compares the signal for the target analyte when NO 1% and UZA are used as the NO^+^/O_2_^+^ source.

#### O_2_^+^ Reagent Ion

As shown in Figure 1, O_2_^+^ is generated along with the NO^+^ reagent ion; therefore, the same voltage settings as the NO^+^/O_2_^+^ reagent ion were used to characterize PFAS using O_2_^+^. The voltage scan performed using O_2_ (UHP) (Figure 1c) shows that at optimized *E*/*N* ∼ 135, O_2_^+^ dominates the reagent ion signal. Since NO^+^ and O_2_^+^ exhibit similar ionization mechanisms, O_2_^+^ may also show sensitivity toward PFAS containing olefinic or vinyl functional groups. This sensitivity could occur via hydride abstraction or charge transfer (R5 and R6).

R5 $$M+O_{2}^{+}\overset{HA}{\to }{\left[M-H\right]}^{+}+\left(O_{2}+H^{-}\right)$$

R6 $$M+O_{2}^{+}\overset{CT}{\to }M^{+}+O_{2}$$

To compare the sensitivity achieved from both O_2_^+^ and NO^+^/O_2_^+^, the calibration was also carried out using O_2_^+^.

### PFAS Sensitivity

To determine the sensitivity for NO^+^/O_2_^+^, O_2_^+^, and H_3_O^+^ under optimized conditions, calibration curves were generated using an 8-component PFAS reference mixture (10.0 ± 0.1) ppm (v/v ratio). The reference mixture was subsequently used for calibration, during which it was diluted with ultrazero air (UZA) to obtain the required calibration concentration. While optimizing the instrument and calibrations, PTFE tubing was used. Comparisons between PFA and PTFE tubing, as well as different tubing lengths of PTFE, showed that the effect of tubing material was negligible (Figure S3).

A five-point calibration was performed by varying the concentration from 19.9 to 99.0 ppb (Figures S4–S10). Calibration curves were obtained by linear regression (least-squares method). Sensitivities were determined from the slopes of the calibration curves, and the limits of detection (LODs) were calculated as 3 times the standard deviation (σ) of the zero-air background (ions/sec) (eq S7).^47^ The LODs were converted to ppb using the sensitivity of the PFAS tested, as described in eq S8. The regression intercept for the calibration of each compound was found to be within the experimental variability and did not significantly affect the accuracy of the quantification. The regression exhibited excellent linearity with a *R* ^2^ = 0.99 for all the calibrations performed. All sensitivities were reported in ions per second per parts per billion by volume (ions sec^–1^ ppbv^–1^), and the LODs were reported in parts per trillion by volume (pptv). It should be noted that, although calibration was performed using mixing ratios in the ppb range, the achieved detection limits are in the ppt range, which are potentially relevant for ambient concentrations of PFAS.

### Calibration Using H_3_O^+^ Reagent Ion

CIMS with H_3_O^+^ has been widely used to characterize VOCs with diversified functional groups.^44,45,48^ To the best of our knowledge, there have been no prior applications reported for H_3_O^+^ CIMS to detect PFAS. In the current study, we found that the poly/perfluoroolefin compounds formed the charged ion via fluoride abstraction (R7) using the H_3_O^+^ reagent ion, detected as [M-19 *m*/*z*], where M is the parent molecule. Likewise, the PVE formed the charged ion via fragmentation of three fluorine atoms (R8), detected as [M-57] with the H_3_O^+^ reagent ion. The sensitivities (ions s^–1^ ppb^–1^) of all tested PFAS using H_3_O^+^ CIMS were lower by 2 orders of magnitude in most cases, compared to NO^+^/O_2_^+^ and O_2_^+^ (Table S2). Targeted PFAS were found to have high LODs in the range from 23 ppt to 2.7 ppb (Table S3). The low sensitivity and high LODs limit the use of H_3_O^+^ as a viable method for the real-time characterization of PFAS in ambient air.

R7 $$C_{n}F_{m}H_{x}\;\text{or}\;C_{n}F_{2n}\to {\left[C_{n}F_{m-1}H_{x}\right]}^{+}\;\text{or}\;{\left[C_{n}F_{2n-1}\right]}^{+}$$

R8 $$C_{n}F_{2n}O\to {\left[C_{n}F_{2n-3}O\right]}^{+}$$

where, *n* represents the number of carbon atoms, m represents number of fluorine atoms, and *x* represents number of hydrogen atoms.

### Calibration Using NO^+^/O_2_^+^ and O_2_^+^ Reagent Ions

In this study, O_2_^+^ was always present alongside NO^+^ during calibrations when using NO as the reagent gas. Since both ionization techniques share similar detection mechanisms for PFAS, calibrations were performed using UZA, NO 1%, and ultrapure O_2_ (O_2_^+^). All PFAS were detected with NO^+^/O_2_^+^ and O_2_^+^ as reagent ions, respectively, with the products as charge transfer (M^+^), hydride abstraction (M – H)^+^, and fluoride abstraction (M – F)^+^. The poly/perfluoroolefins were detected as hydride abstraction or fluoride abstraction along with charge transfer ions (R9 & R10). In contrast, the PVEs formed charged ions through the charge transfer process (R11). Perfluoroalkane was detected as a hydride abstraction ion (R9).

R9 $$C_{n}F_{m}H_{x}\;\text{or}\;C_{n}F_{2n}\to {\left[C_{n}F_{m}H_{x-1}\right]}^{+}\;\text{or}\;{\left[C_{n}F_{2n-1}\right]}^{+}$$

R10 $$C_{n}F_{m}H_{x}\;\text{or}\;C_{n}F_{2n}\to {\left[C_{n}F_{m}H_{x}\right]}^{+}\;\text{or}\;{\left[C_{n}F_{2n}\right]}^{+}$$

R11 $$C_{n}F_{2n}O\to {\left[C_{n}F_{2n}O\right]}^{+}$$

where *n* represents the number of carbon atoms, *m* represents the number of fluorine atoms, and *x* represents the number of hydrogen atoms.

The probability of a molecule undergoing charge transfer or fragmentation to form a charged ion depends on whether the ionization energy (IE) of the molecule is less than or greater than the recombination energy (RE) of the reagent ion. For a charge transfer to occur, the RE of the NO^+^/O_2_^+^ reagent ion must be greater than or equal to the IE of the neutral PFAS molecule.^41^ The RE of NO^+^ is 9.5 eV,^43^ while that of O_2_^+^ is about 12 eV,^36^ whereas the IE of per/polyfluoroolefins is in the range of 10.5–11.5 eV.^49,50^ The PVEs have ionization energies in the range of 9–10 eV.^51,52^ Given that the RE of O_2_^+^ is greater than the IE of per/poly fluoro olefins, O_2_^+^ is more likely to form a charged ion through charge transfer, but a charge transfer process from either NO^+^ or O_2_^+^ reagent ions is possible. Per/poly fluoroolefins also form charged ions through dissociative ionization (DI), where DI is the simultaneous ionization and fragmentation of a molecule to form a charged species. The dissociation energy (DE) of the C–F bond is typically around 5.4–5.5 eV.^53^ It is safe to assume that the energy transferred during the collision with the reagent ion could result in the cleavage of one of its C–F bonds, leading to DI. Given that O_2_^+^ and NO^+^ were both present as reagent ions during the calibrations, the formation of charged ions through CT and DI happened simultaneously. HF-alkane has an ionization energy of ∼13 eV,^54^ so it produces charged ions predominantly through hydride abstraction.

All eight PFAS tested (i.e., HFO-1234yf, PFP, HFP, PFB, PFPe, PMVE, PEVE, and PPVE) exhibited higher sensitivity and lower LODs using both NO^+^/O_2_^+^ and O_2_^+^ reagent ions compared to the H_3_O^+^ reagent ion. Figure 3 and Table S3 present an intercomparison of sensitivity across reagent ions, including NO^+^/O_2_^+^ from the two sources (UZA and NO 1%). The sensitivity trend was as follows: O_2_^+^ > NO^+^/O_2_^+^(NO 1%) > NO^+^/O_2_^+^ (UZA) > H_3_O^+^. Among the PFAS analytes, fluoroolefin (FO) compounds demonstrated one of the highest sensitivities to O_2_^+^ and NO^+^/O_2_^+^. When comparing both charge transfer ion or hydride/fluoride abstraction ion within the per/polyfluoroolefin group, HFP had the highest sensitivity, followed by HFO-1234yf, PFB, and PFPe. For the CT ion HFP had a sensitivity of 3730 ± 60 ions s^–1^ ppb^–1^ in O_2_^+^, 460 ± 10 ions s^–1^ ppb^–1^ in NO^+^/O_2_^+^ (NO 1%), and 330 ± 10 ions s^–1^ ppb^–1^ (NO^+^/O_2_^+^, UZA). For PVE compounds (CT ion), the sensitivity order was PEVE > PPVE > PMVE in both NO^+^/O_2_^+^and O_2_^+^ reagent ions.

**Figure 3:**
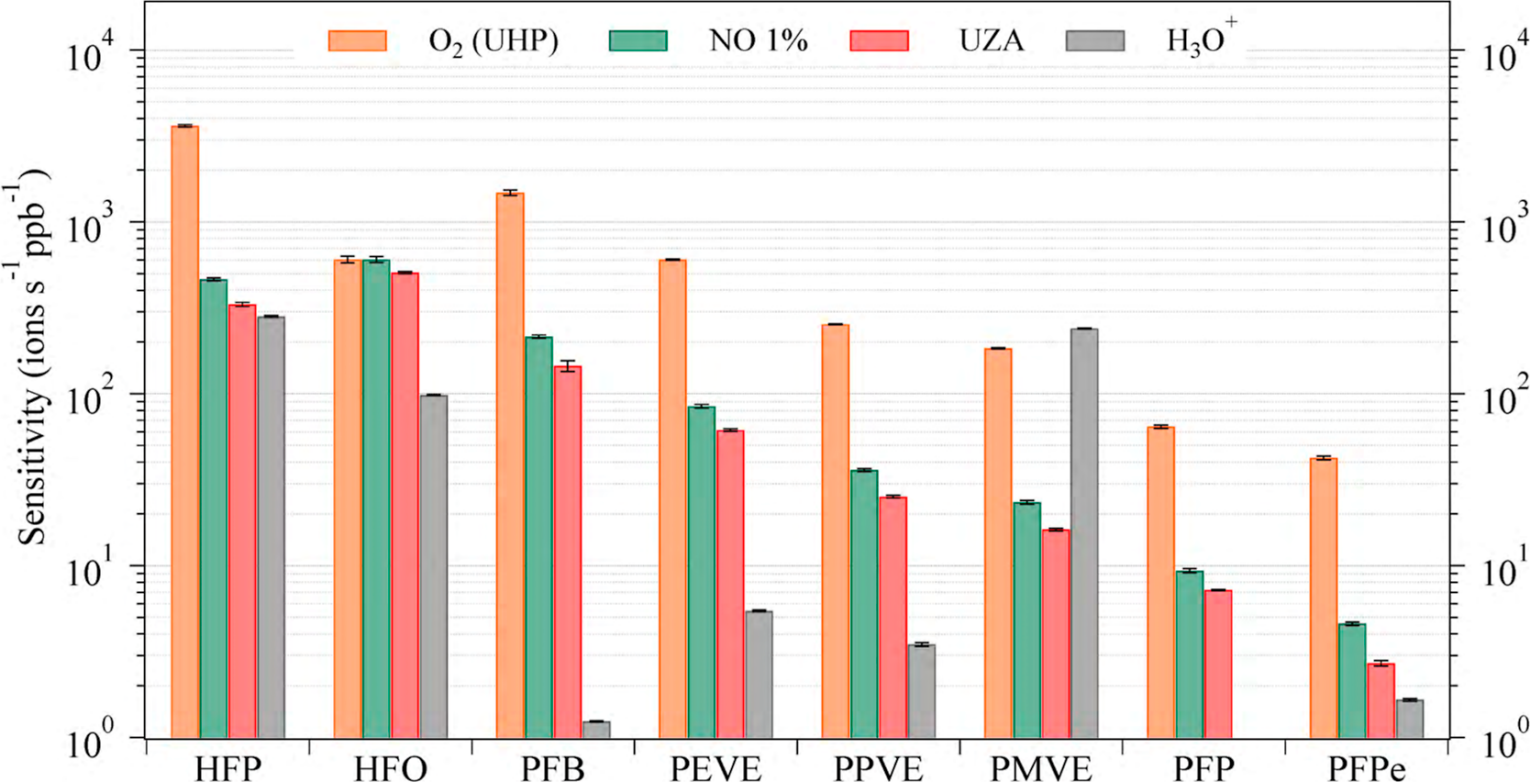
Intercomparison between sensitivities of PFAS measured in NO^+^/O_2_^+^, O_2_^+^, and H_3_O^+^ reagent ions using CIMS. The *y*-axis is in log scale. For HFP, PFB, PEVE, PPVE, PMVE, and PFPe with NO^+^/O_2_^+^ and O_2_^+^ reagent ion sensitivities for the charge transfer ions (M^+^) are plotted. For HFO and PFP sensitivity for the hydride abstraction ion (M – H)^+^ are plotted. For the H_3_O^+^ reagent ion, (M – F)^+^ ions are plotted for HFP, HFO (representing HFO-1234yf), PFB, and PFPe. For PEVE, PPVE, and PMVE, (M – 3F)^+^ are plotted. PFP is not detected with the H_3_O^+^.

The low background for targeted PFAS with NO^+^/O_2_^+^and O_2_^+^ reagent ions correspond to low LODs for the targeted PFAS (Table S3 and Figure 4). The LOD for per/polyfluoroolefins (CT ions) ranged from 2 to 6 ppt, except for PFPe, with an LOD that ranged from 9.5–55.4 ppt, depending on the reagent ion used. For PVE compounds, LODs are in the range of 5–25 ppt. PFP compounds, which were only able to be detected by NO^+^/O_2_^+^ and O_2_^+^, showed LODs of 18 ppt (O_2_^+^), 102 ppt (NO^+^/O_2_^+^, NO 1%), and 89.2 ppt (NO^+^/O_2_^+^, UZA).

**Figure 4:**
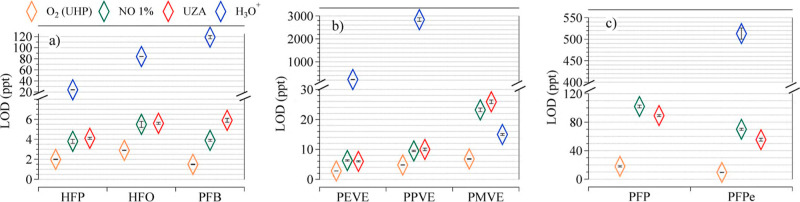
Intercomparisons between the LODs of PFAS measured in NO^+^/O_2_^+^, O_2_^+^, and H_3_O^+^ reagent ions using CIMS. (a) intercomparison for HFP, HFO and PFB. (b) intercomparison for PEVE, PPVE, and PMVE. (c) intercomparison for PFP and PFPe. For HFP, PFB, PEVE, PPVE, PMVE, and PFPe with NO^+^/O_2_^+^ and O_2_^+^ LODs (10 s) for the charge transfer ions (M^+^) are plotted. For HFO and PFP LODs (10 s) for the hydride abstraction ion (M – H)^+^ are plotted. For the H_3_O^+^ reagent ion, (M – F)^+^ ions are plotted for HFP, HFO, PFB, and PFPe. For PEVE, PPVE, and PMVE, (M – 3F)^+^ are plotted. PFP is not detected with the H_3_O^+^. The yellow, green, red, and blue colors each represent the reagent ion with O_2_ (UHP), NO (1%), UZA, and H_3_O^+^ respectively.

In addition, while all perfluorinated compounds tested had high LODs but were detectable using H_3_O^+^ reagent ion, they exhibited high sensitivity and low LODs with NO^+^/O_2_^+^ and O_2_^+^ reagent ions. It is also worth noting that iodide-adduct CIMS and acetate–CIMS, which has been widely used to characterize PFAS,^3,31,33^ are insensitive to the PFAS analytes targeted in the current study,^32,33^ making the positive reagent ion methodology unique for quantification of olefin and alkane PFAS species in real-time. Given that most of the compounds detected with NO^+^/O_2_^+^ and O_2_^+^ reagent ion have an olefin group, this method has the potential to be applied to other PFAS with an olefin group beyond those tested in this study.

### Real-Time Measurement of HFO-1234yf

To demonstrate the potential for real-time measurement of PFAS using positive ion mode with NO^+^/O_2_^+^ reagent chemistry, we simulated a workplace exposure to fugitive emissions using a commercially available HFO-1234yf gas cartridge. To simulate exposure, the charging hose of the refrigerant gas cartridge was briefly punctured for a fraction of a second, generating short puffs of gas. To minimize direct exposure, the cartridge was handled in a well-ventilated area while wearing a 3M respirator mask. Several 1–2 s puffs were generated in a well-ventilated laboratory at a distance ranging from 0.9 to 6.4 m from the instrument inlet, as illustrated in Figure S10, simulating fugitive emissions when a refrigerant is connected to an automobile to recharge the refrigerant. Although the puff at 6.4 m was generated inside the fume hood to reduce ambient concentrations, a change in HFO-1234yf was still observed by the CIMS. A sharp increase in the signal for HFO-1234yf was observed when each puff was generated, followed by a dilution decay, as seen in Figure 4. It is important to note that puff volumes at each distance may not be identical due to the short duration of uncontrolled release, and therefore, the observed signal intensity should not be directly correlated with distance. Instead, peak intensities are more strongly influenced by puff volume and may exhibit uncertainties arising from variability in the generation of each puff. The high time resolution (1 s) of the CIMS enabled the detection of rapid temporal changes in HFO-1234yf concentrations during the experiment, as demonstrated in Figure 5. Concentrations quantified using the HFO-1234yf sensitivity ranged from approximately 500–3000 ppb. These levels were well above the method’s limit of detection (LOD: 5.1 ppt) and limit of quantification (LOQ: 16.9 ppt), despite the high laboratory air exchange rate of 7.6 h^–1^ (see Supporting Information Section S4 for details on air exchange rate determination), suggesting that workplace exposure of HFO-1234yf could be potentially significant as demonstrated by this real-time detection analytical method. Rapid decay of HFO-1234yf was observed during the perturbation experiment at a laboratory air exchange rate of 7.6 h^–1^(Figure 5). Given its much lower global warming potential compared to sulfur hexafluoride (SF_6_),^55,56^ HFO-1234yf, when measured using the developed CIMS method, may serve as a suitable alternative tracer for determining indoor air exchange rates. The potential of the developed method in identifying the leak rates could also be crucial in constraining the atmospheric burden of similar PFAS. It will also enable estimation of the production and deposition of their degradation products.

**Figure 5:**
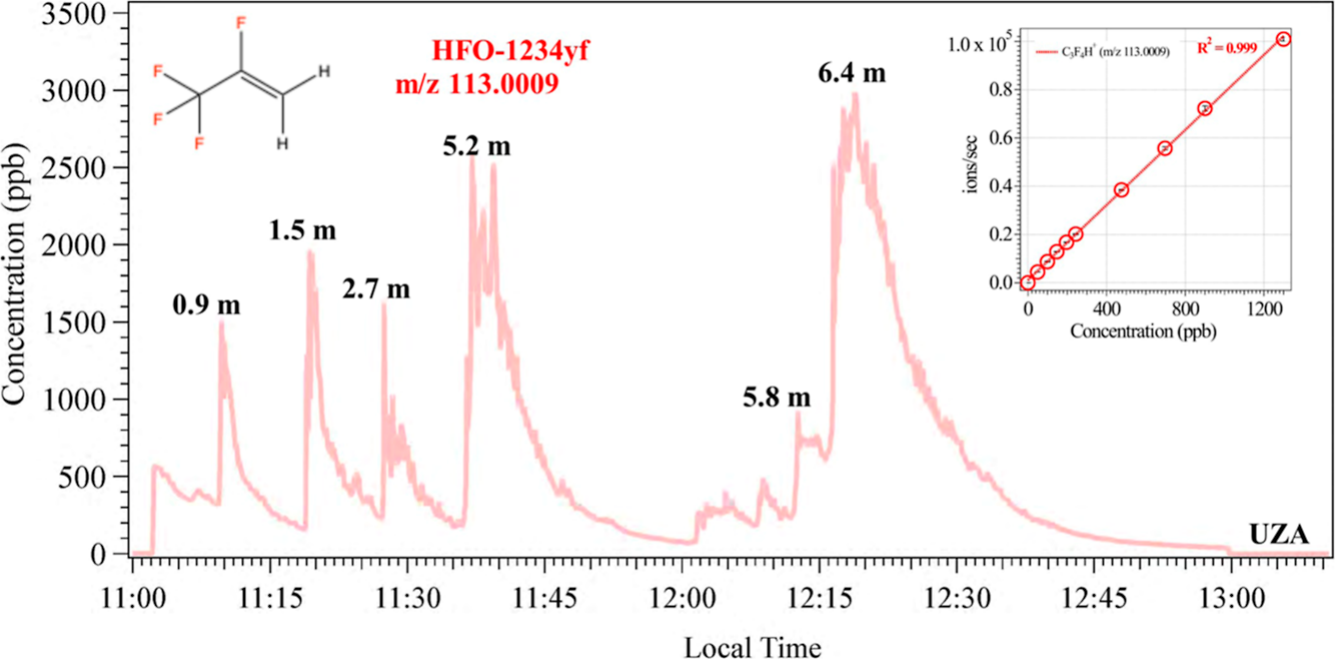
Real-time measurement for HFO-1234yf (*m*/*z* 113.0009). Each spike in the time series represents a puff generated from the HFO-1234yf cartridge. The distance (*m*) of the puff from the CIMS inlet is listed on top of the corresponding spikes. The spike intensity correlated with puff volume generated rather than the distance from the instrument. The inset shows the chemical structure of HFO-1234yf and the linearity plot for concentrations as high as 1300 ppb.

### Atmospheric Implications

The current study provides real-time quantification and characterization of HFO-1234yf, HF-alkane, PMVE, PEVE, PPVE, HFP, PFB, and PFPe by using CIMS operated with NO^+^/O_2_^+^, O_2_^+^, and H_3_O^+^ reagent ions for the first time. Key PFAS tested in this study, such as HFP, PFB, PFP, PMVE, PEVE, and PPVE, may have high concentrations near manufacturing facilities,^34^ potentially allowing for real-time detection using the CIMS with NO^+^/O_2_^+^and O_2_^+^ reagent ions near major point sources. The technique may also be extended to study poorly constrained leaks in buildings, grocery stores, or workplace exposure to refrigerants such as HFO-1234yf and related health impacts. The developed method may also be crucial in estimating the atmospheric burden and emissions of gas-phase PFAS near known point sources.

A recent study modeled the emissions of PFO and PVE near a known point source to be 1290 kg yr^–1^ to 40,000 kg yr^–1^, but their concentrations in the atmosphere are poorly constrained.^28,34^ Their high volatility and insolubility in water likely prevent PFO, PVE, and HF-alkane from partitioning to aerosol particles or undergoing wet deposition.^28,34^ Previous studies using the Goddard Earth Observing System-Chemistry (GEOS-Chem) model estimate the annual global mean concentration of HFO-1234yf to be 0.34 pptv.^56,57^ D’Ambro et al.^34^ predicted that 80% of total PFAS emissions near a known manufacturing source were from three compounds, namely tetrafluoroethylene (TFE), hexafluoropropylene oxide (HFPO), and HFP, despite unknown exposure and deposition rates. The ppt-level detection limits of the above PFAS species suggest that real-time quantification of these compounds using the NO^+^/O_2_^+^ and O_2_^+^ reagent ions is achievable in certain ambient environments, especially near their emission sources.

Due to the reactivity of the olefin bond against hydroxyl radical, the atmospheric lifetime of PFO and PVE is estimated to be very short (a few hours to about a week),^56,58^ making real-time measurement crucial. Additionally, the targeted PFAS may not partition to aerosol particles or undergo wet deposition,^28,34^ but may act as precursors that can produce more toxic PFAS oxidation products.^59–61^ For instance, HFO-1234yf has been shown to produce TFA which is highly soluble in aqueous phases,^62,63^ is known to have phytotoxicity and possesses environmental risks.^62^ Recently, high concentrations of TFA (in a range of 0.3 ng L^–1^ to 148 ng L^–1^) along with other short-chain PFCAs have been reported in the Arctic ice cores.^23^ A recent study reported seasonal variations in short-chain PFCAs in ambient air, with higher concentrations observed during summer, ranging from 0.01 to 0.9 pptv. A recent study with offline sampling (∼48 h time integration) also reported the PFCA concentrations both in particles (28.8–206 pg/m^3^) and gas phase (1.6–61.4 pg/m^3^), in the urban atmosphere, with the possibility of dynamic exchange between particle and gas phase.^64^ The increase in PFCA concentrations may be attributed to enhanced photooxidation, which can only be fully understood through parallel monitoring of precursor emissions. Monitoring persistent short-chain PFCA precursors can provide valuable insight into their emissions and atmospheric chemistry, which in turn may help constrain their representation in global models. The NO^+^/O_2_^+^ or O_2_^+^ CIMS can potentially be deployed in combination with techniques such as acetate–CIMS and I^–^–CIMS to investigate precursors and oxidation products, thereby enabling a more comprehensive understanding of the chemical evolution of gas-phase PFAS.

Given their potential for atmospheric emission, transformation and impacts, the ability to measure the atmospheric dynamics of the above PFAS using highly sensitive real-time measurement methods like HR-ToF-CIMS with NO^+^/O_2_^+^and O_2_^+^ may have significant advantages. The present study demonstrates that O_2_^+^ is the most sensitive reagent ion for detecting the targeted PFAS, followed by NO^+^/O_2_^+^. In contrast, the relatively high LODs obtained with the traditional H_3_O^+^ reagent ion suggest its unsuitability for ambient measurements. Considering that gas-phase PFAS are likely present at very low concentrations (ppt to ppq levels),^11^ achieving low detection limits in the ppt range may allow the use of NO^+^/O_2_^+^or O_2_^+^ for ambient monitoring. To the best of our knowledge, no prior estimated emissions through real-time ambient measurement have been reported for the targeted class of gas-phase PFAS. The analytical approach developed in the current study has the potential to enable the detection of fugitive emissions, improve emission factor estimates, and advance the understanding of the chemical evolution, partitioning, and fate of PFAS in ambient air. Even though the highest sensitivity and lowest LODs are achieved with O_2_^+^, we recommend using NO^+^/O_2_^+^ mixed ionization for ambient measurement, given that with pure O_2_^+^ (Figure 1c), O_2_^+^ dominates, which is a harder ionization technique (IE ∼ 12.07 eV) and may result in more fragmentation, making the ambient data processing complex.^43^

## Supplementary Material



## Notes

The Supporting Information includes Section S1: preparation of a 10 ppm reference mixture of PFAS; Figure S1 chemical structures of the gas phase PFAS standards; Table S1: 14-component reference mixture for volatile organic compounds (VOCs) in ultrapure nitrogen (Apel-Riemer Environmental, Inc. Miami, Florida); Section S2 calculation of *E*/*N*; Figure S2 difference in the sensitivity for 2,3,3,3-tetrafluoropropene (HFO-1234yf) tested at BSQ voltage 380 V (green), 235 V (red), and 150 V (blue); Figure S3 comparing tube passivation (PFA vs PTFE) and different tubing length for PTFE; Figure S4 calibration plot for PFAS in PTR mode; Figure S5 calibration plot for HFO, where the signal intensities in ions/sec are plotted as a function of concentration (ppb); Figure S6 calibration plot for HFP, where the signal intensities (ions/sec) are plotted as a function of concentration (ppb); Figure S7 calibration plot for PFB, where the signal intensities (ions/sec) are plotted as a function of concentration (ppb); Figure S8 calibration plot for PFPe, where the signal intensities (ions/sec) are plotted as a function of concentration (ppb); Figure S9 calibration plot for FVE, where the signal intensities (ions/sec) are plotted as a function of concentration (ppb); Figure S10 calibration plot for PFP, where the signal intensities (ions/sec) are plotted as a function of concentration (ppb); Section S3 calculation of calibration gas concentration and limit of detection; Table S2 intercomparisons of sensitivity of selected PFAS using NO^+^, O_2_^+^, and H_3_O^+^ reagent ions, respectively; Table S3 intercomparisons of the LOD (10 s) and LOQ of selected PFAS using the NO^+^/O_2_^+^, O_2_^+^, and H_3_O^+^ reagent ions, respectively; Figure S11 schematic representation for the real-time measurement for R-1234yf; Section S4 determination of air exchange rate (PDF)
