# Supplementary material for: Real-Time Identification and Quantification of Per- and Polyfluoroalkyl Substances Using High-Resolution Time-of-Flight Chemical Ionization Mass Spectrometry with Positive Reagent Ions
Source: Anal Chem. 2025 Dec 25;98(1):124–33. doi: 10.1021/acs.analchem.5c02489 (PMC12809635; doi:10.1021/acs.analchem.5c02489)
Supplement: Supplementary file 1 [file ac5c02489_si_001.pdf]

Supporting Information

Real-Time Identification and Quantification of Per- and Polyfluoroalkyl Substances (PFAS) Using High-Resolution Time-of-Flight Chemical Ionization Mass Spectrometry (HR-ToF-CIMS) with Positive Reagent Ions

Sahir Gagan[1], Miska Olin[1], Alana J. Dodero[1], Siddharth Gopalakrishnan[1], Sining Niu[1], Michael J. Davern[2], Barbara J. Turpin[3], Jason D. Surratt[2,3], Yue Zhang[1*]*

[1]Department of Atmospheric Sciences, Texas A&M University, College Station, TX, USA 77843

[2] Department of Chemistry, College of Arts and Sciences, University of North Carolina at Chapel Hill, Chapel Hill, North Carolina, USA 27514

[3]Department of Environmental Sciences and Engineering, Gillings School of Global Public Health, University of North Carolina at Chapel Hill, Chapel Hill, North Carolina, USA 27599

October 2025

Analytical Chemistry

[*]*Corresponding authors:* Yue Zhang, yuezhang@tamu.edu

Jason D. Surratt, surratt@unc.edu

No. of pages: 23

No. of figures: 11

No. of tables: 3

Table of Contents

S1. Preparation of a 10-ppm reference mixture of PFAS compounds....................................S3

Figure S1. Chemical structures of the gas-phase PFAS standards. S5

Table S1. 14-component reference mixture for VOCs in ultrapure nitrogen S6

S2. Calculation of E/N S7

Figure S2. Difference in the sensitivity for HFO-1234yf tested at different BSQ voltage S8

Figure S3. Comparing PFA vs PTFE, and different tubing length...S9

Figure S4. The figure shows the instrument sensitivity with H_3O^+ as the reagent ion, a) shows the results of per/polyfluoroolefins..S10

Figure S5. Calibration plot for HFO. S11

Figure S6. Calibration plot for HFP...S12

Figure S7. Calibration plot for PFB S13

Figure S8. Calibration plot for PFPe...S14

Figure S9. Calibration plot for VE..S15

Figure S10. Calibration plot for PFP S16

S3. Calculation of calibration gas concentration and limit of detection S17

Table S2. Intercomparisons of the sensitivity (ions s^{-1} ppb^{-1}) of selected PFAS S18

Table S3. Intercomparisons of the LOD (10 s) and LOQ of selected PFAS species using the NO^+/O_2^+, O_2^+, and H_3O^+ reagent ion. S20

Figure S11. Schematic representation for the real-time measurement of HFO-1234yf S22

S4. Determination of Air Exchange Rate S23

S1. Preparation of a 10-ppm reference mixture of PFAS compounds

Using pure standards, a 10 ppm PFAS reference mixture was prepared at Nutech Instruments, Plano, TX, USA. The mixture was created through a two-step dilution process: first, a 100-ppm reference mixture was prepared, which was then diluted to achieve the final 10-ppm concentration. The dilution calculation is provided in Eq. S1.

$$C_1 V_1 = C_2 V_2 \qquad \text{(S1)}$$

Where C_1 is the concentration of the PFAS compound in pure standard, V_1 is the volume of the pure standard required for the target concentration, V_2 is the final volume of the reference mixture at 40 psi, and C_2 is the target concentration.

$$C_1 = \frac{10^6\ ppm \times purity}{\frac{V_{calc}}{V_{Total}}} \qquad \text{(S2)}$$

Where

$$V_{calc} = \frac{Molecular\ weight\ of\ PFAS\ standard\ (\frac{g}{mol})}{Net\ Weight\ of\ Pure\ Standard\ (g)} \times Volume\ of\ gas\ at\ STP\ \left(\frac{L}{mol}\right) \times 1000 \qquad \text{(S3)}$$

V_{calc} is the volume of pure PFAS standard in the low-pressure gas cylinder calculated using volume of gas at STP.

$$V_{Total} = V_{calc} + Volume\ of\ cylinder\ for\ pure\ standard \qquad \text{(S4)}$$

$$V_2 = Canister\ volume\ (L) \times 1000 \times \frac{Final\ Pressure - Vacuum\ Pressure}{abs(Vacuum\ pressure)} \qquad \text{(S5)}$$

An example calculation is shown below for one of the PFAS standards, 2,3,3,3-terafluoropropene (HFO-1234yf)

Step 1: Preparation of 50 ppm (v/v) PFAS reference standard balanced by N_2

Molecular wt. of HFO-1234yf = 114.04

Purity = 0.99

Net weight of the pure standard = 25g

Volume of the gas cylinder = 485 ml

Volume of the gas at STP = 24.45 L/mol

Target Concentration (C_2) = 50 ppm

Volume of the canister = 6 L

Final Pressure = 30 psi

Vacuum Pressure = -14.7 psi

We used the relation $C_1V_1 = C_2V_2$ for the dilution. C_1 (ppm) is the concentration of HFO-1234yf (purity = 99%), which is calculated based on the actual volume of the PFAS standard in the cylinder (V_{total}), volume of pure standard (V_{calc}) at STP conditions, and purity of the PFAS standard.

$$V_{calc} = \frac{114.04\,(\frac{g}{mol})}{25\,(g)} \times 24.45\left(\frac{L}{mol}\right) \times 1000\,(\frac{ml}{L})$$

$$V_{calc} = 5359.96\,ml$$

Total Volume of the PFAS standard in a 485 ml gas cylinder is calculated using V_{total}

$$V_{Total} = V_{calc} + Volume\ of\ cylinder\ for\ pure\ standard$$

$$V_{Total} = 5359.96\,ml + 485\,ml = 5844.96\,ml$$

$$C_1 = \frac{10^6\,ppm \times purity}{\frac{V_{calc}}{V_{Total}}}$$

$$C_1 = \frac{10^6\,ppm \times 0.99}{\frac{5359.96}{5844.96}} = 907852\,ppm$$

Final Volume of 6L canister (V2) is calculated using the equation:

$$V_2 = Canister\ volume\ (L) \times 1000\frac{ml}{L} \times \frac{Final\ Pressure\ (psi) - Vacuum\ Pressure(psi)}{abs(Vacuum\ pressure)}$$

$$V_2 = 6\,(L) \times 1000\frac{ml}{L} \times \frac{30\,psi - (-14.7\,psi)}{abs(-14.7\,psi)}$$

$$V_2 = 18245\,ml$$

The volume of pure PFAS standard required *(V_1)* to make 50 ppm reference standard

$$C_1 V_1 = C_2 V_2$$

$$907852\,ppm \times V_1 = 50\,ppm \times 18245\,ml$$

$$V_1 = 0.995\,ml$$

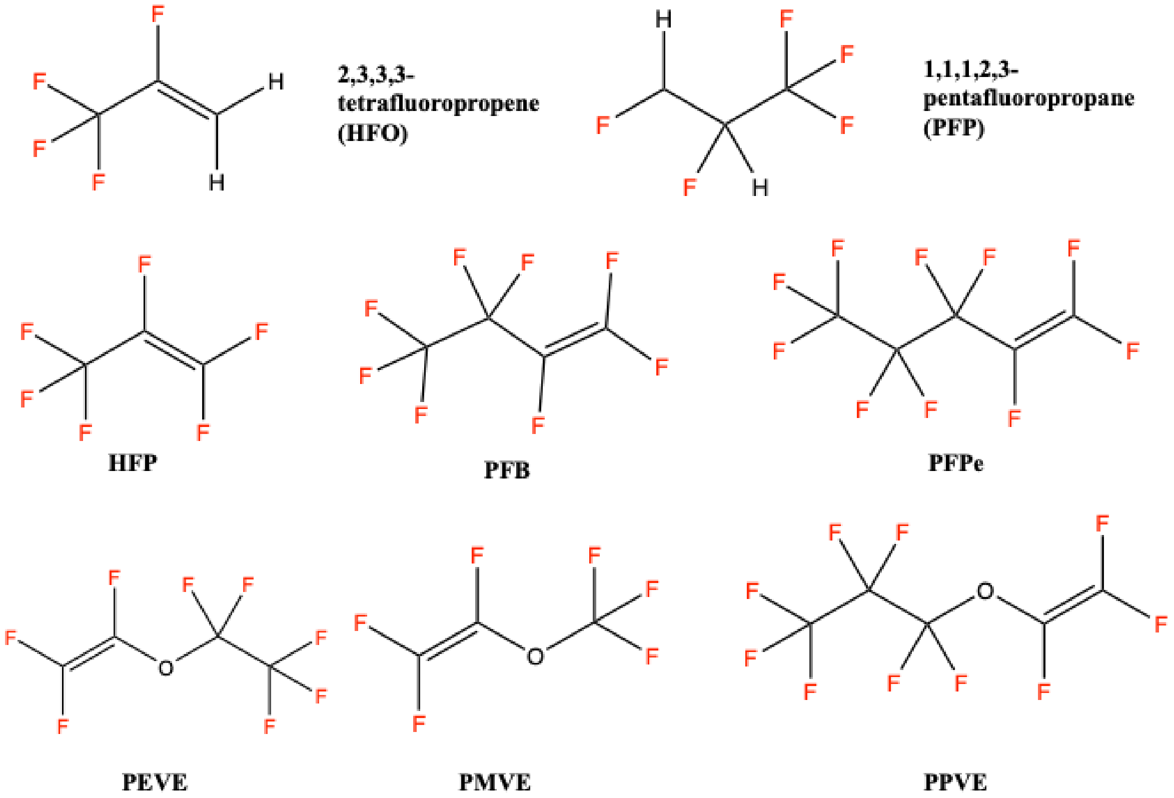

Figure S1. Chemical structures of the gas-phase PFAS standards.

Table S1. 14-component reference mixture for volatile organic compounds (VOCs) in ultrapure nitrogen (Apel-Riemer Environmental, Inc. Miami, Florida).

S.No	Standard Compound	CAS Number
1	Acetonitrile	70-05-8
2	Ethanol	64-17-5
3	Acrylonitrile	107-13-1
4	Acetone	67-64-1
5	Isoprene	78-79-5
6	Methyl Ethyl Ketone	78-93-3
7	Benzene	71-43-2
8	m-xylene	108-38-3
9	1,2,4-Trimethylbenzene	95-63-6
10	α-pinene	80-56-8
11	Octametylcyclotetrasiloxane	556-67-2
12	Decametylcyclopentasiloxane	541-02-6
13	Methyl Vinyl Ketone	78-94-4
14	β-Caryophyllene	87-44-5

S2. Calculation of E/N

The reduced electric field (E/N, Townsends (Td)), defined as the ratio of the electric field strength (E, V/cm) to the neutral gas number density (N, molecules/cm^3), was calculated using the FIMR (Focusing Ion-Molecule Reactor) operating parameters. Specifically, E/N (Td) was derived from the applied FIMR front and back voltages, reactor length (L, 10 cm), pressure (2.2 mbar), and reactor temperature (100 °C). The N was calculated using the ideal gas law under the given pressure and temperature conditions. The effective voltage (volts, V) across the reactor was determined from the difference between the front and back voltages. The E/N (Td) value was then obtained by dividing the electric field (V/L) by the number density (N), as shown in Equation S9.

$$\frac{E}{N} \sim \frac{\Delta V/L}{P/T} \qquad \text{(S6)}$$

It is important to note that the Vocus PTR (Proton Transfer Reaction) instrument employs a combination of radio frequency (RF) and direct current (DC) fields to facilitate the chemical ionization of analyte molecules. The presence of an RF field introduces additional complexity to the E/N calculation, as the RF contributes energy in the radial direction and its effects are mass-dependent. Therefore, while the calculated E/N provides an estimate of the axial electric field strength normalized to gas density, it does not fully account for the additional energy imparted by the RF field.

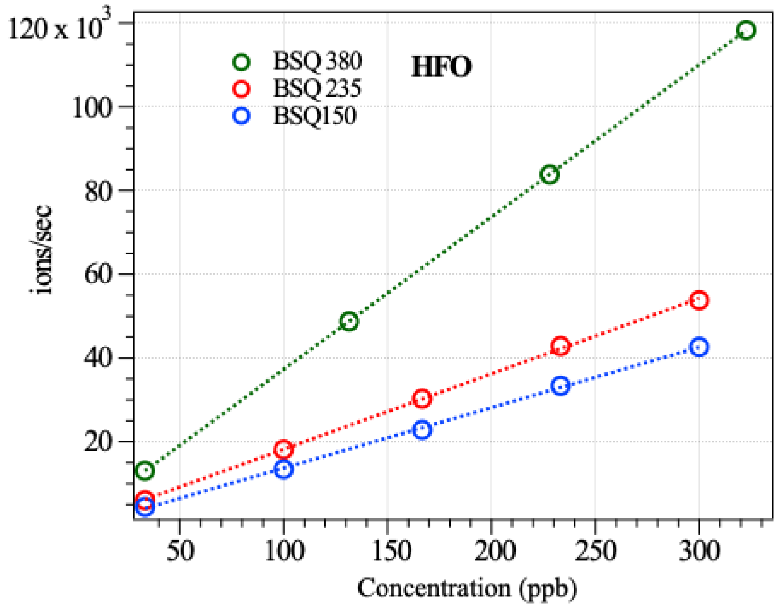

Figure S2**.** Difference in the sensitivity for 2,3,3,3-tetrafluoropropene (HFO-1234yf, denoted as HFO) tested at BSQ voltage 380V (green), 235V (red), and 150V (blue).

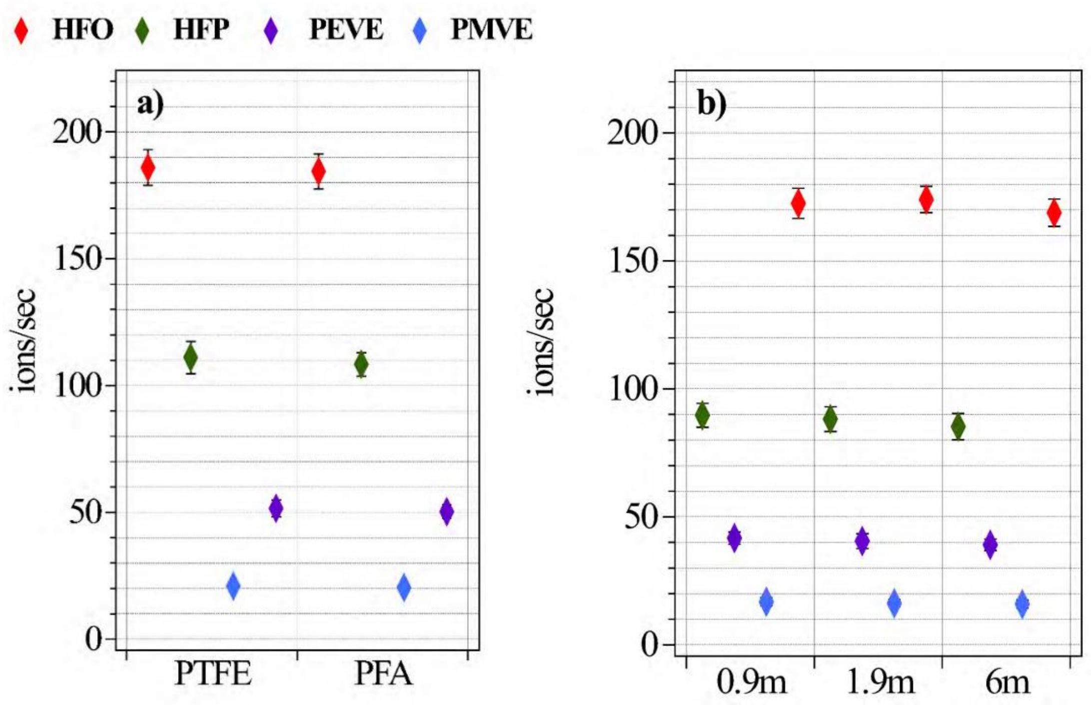

Figure S3. a) Comparing the passivation of target PFAS between PTFE and PFA tubing. b) Comparing the signal (ions/sec) at different lengths of 0.9m, 1.9 m, and 6 m for PTFE tubing.

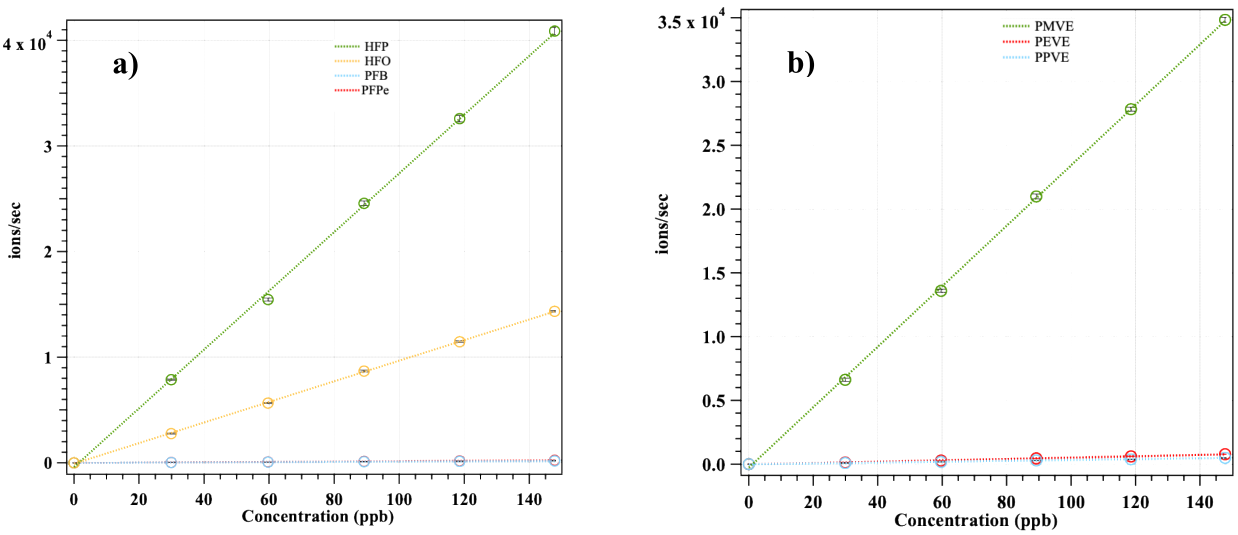

Figure S4. The figure shows the instrument sensitivity with H_3O^+ as the reagent ion, **a)** shows the results of per/polyfluoroolefins. **b)** shows the results of fluoro-vinyl ethers. The linear regression lines were used to derive sensitivity. The error bars represent the standard deviation for ions/sec and fall within the markers for each data point.

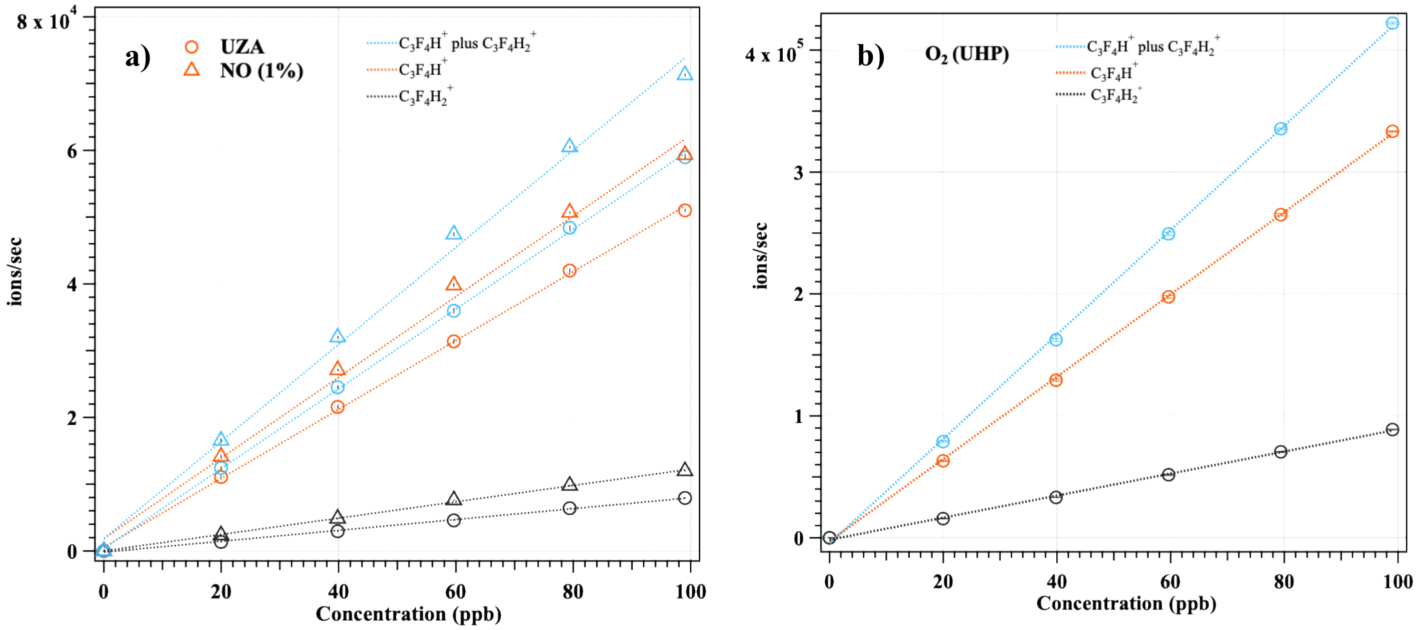

Figure S5. Calibration plot for HFO-124yf, where the signal intensities in ions/sec are plotted as a function of concentration (ppb). **a)** shows the result for instrument sensitivity with NO^+/O_2^+ as the reagent ion, the circle represents UZA, and the triangle represents NO 1% as a source of the reagent ion. **b)** shows the result for instrument sensitivity with O_2^+ reagent ion, O_2 (UHP) is used as source for the reagent ion. The linear regression lines were used to derive sensitivity. The error bars represent the standard deviation for ions/sec and fall within the markers for each data point.

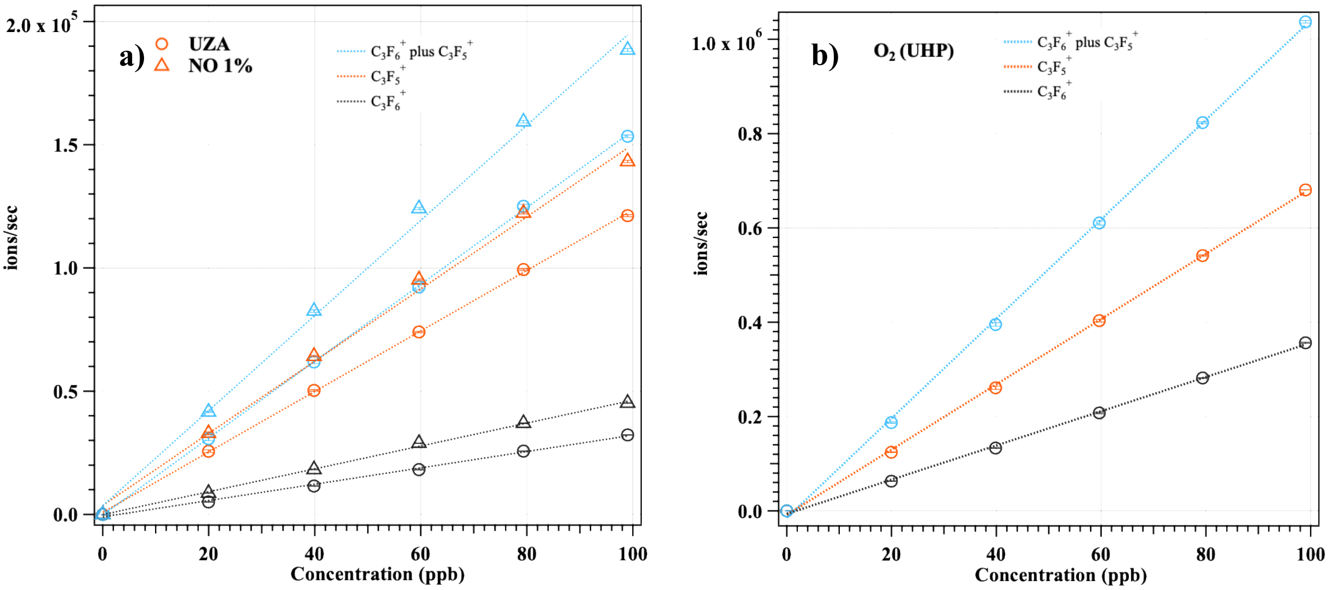

Figure S6. Calibration plot for HFP, where the signal intensities in ions/sec are plotted as a function of concentration (ppb). **a)** shows the result for instrument sensitivity with NO^+/O_2^+ as the reagent ion, the circle represents UZA, and the triangle represents NO 1% as a source of the reagent ion. **b)** shows the result for instrument sensitivity with O_2^+ reagent ion, O_2 (UHP) is used as source for the reagent ion. The linear regression lines were used to derive sensitivity. The error bars represent the standard deviation for ions/sec and fall within the markers for each data point.

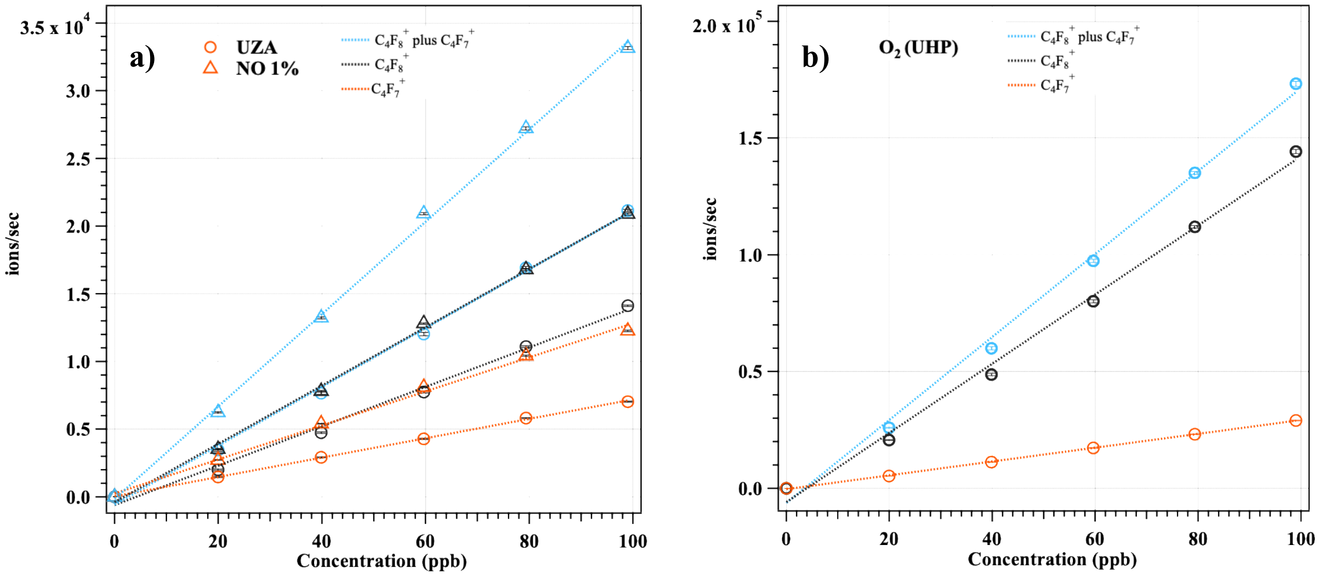

Figure S7. Calibration plot for PFB, where the signal intensities in ions/sec are plotted as a function of concentration (ppb). **a)** shows the result for instrument sensitivity with NO^+/O_2^+ as the reagent ion, the circle represents UZA, and the triangle represents NO 1% as a source of the reagent ion. **b)** shows the result for instrument sensitivity with O_2^+ reagent ion, O_2 (UHP) is used as source for the reagent ion. The linear regression lines were used to derive sensitivity. The error bars represent the standard deviation for ions/sec and fall within the markers for each data point.

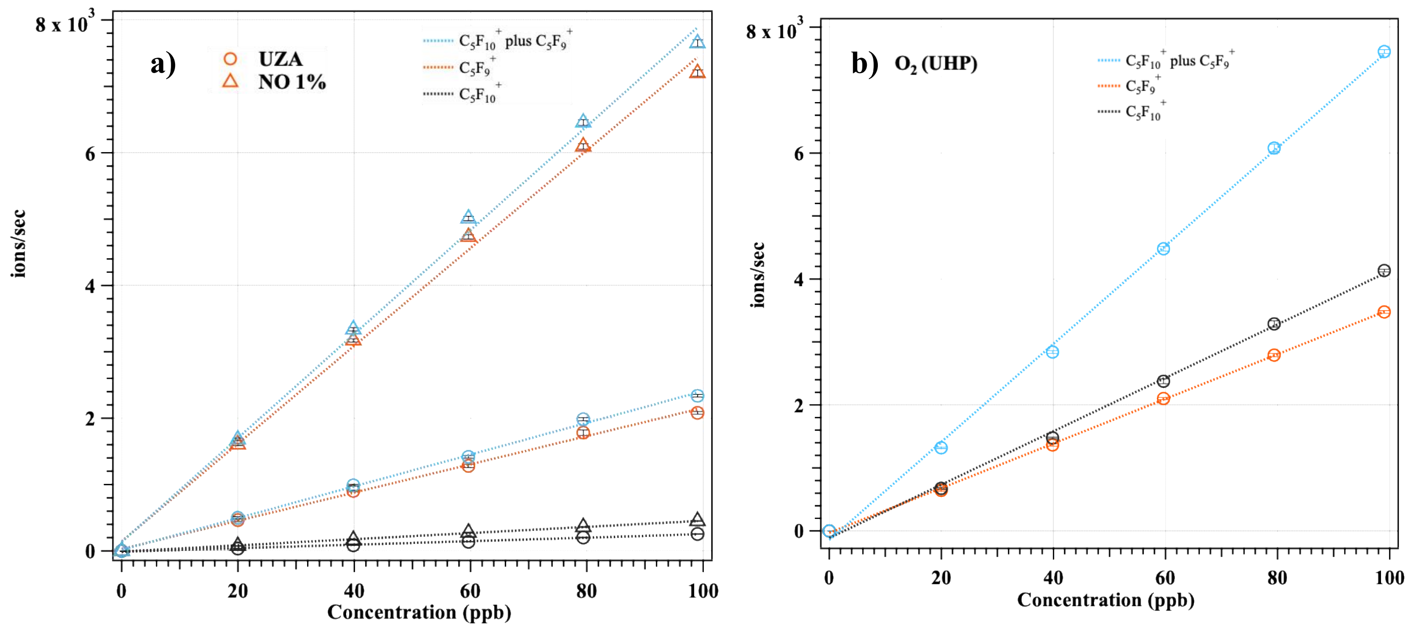

Figure S8. Calibration plot for PFPe, where the signal intensities in ions/sec are plotted as a function of concentration (ppb). **a)** shows the result for instrument sensitivity with NO^+/O_2^+ as the reagent ion, the circle represents UZA, and the triangle represents NO 1% as a source of the reagent ion. **b)** shows the result for instrument sensitivity with O_2^+ reagent ion, O_2 (UHP) is used as source for the reagent ion. The linear regression lines were used to derive sensitivity. The error bars represent the standard deviation for ions/sec and fall within the markers for each data point.

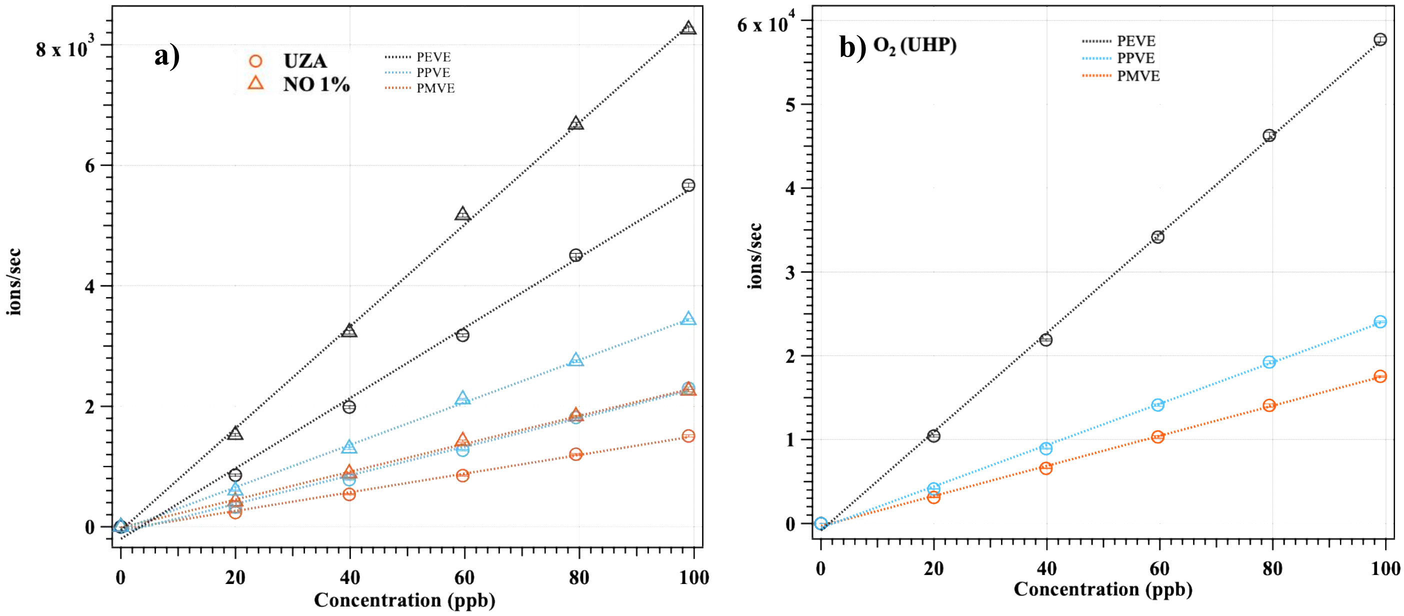

Figure S9. Calibration plot for VE, where the signal intensities in ions/sec are plotted as a function of concentration (ppb). **a)** shows the result for instrument sensitivity with NO^+/O_2^+ as the reagent ion, the circle represents UZA, and the triangle represents NO 1% as a source of the reagent ion. **b)** shows the result for instrument sensitivity with O_2^+ reagent ion, O_2 (UHP) is used as source for the reagent ion. The linear regression lines were used to derive sensitivity. The error bars represent the standard deviation for ions/sec and fall within the markers for each data point.

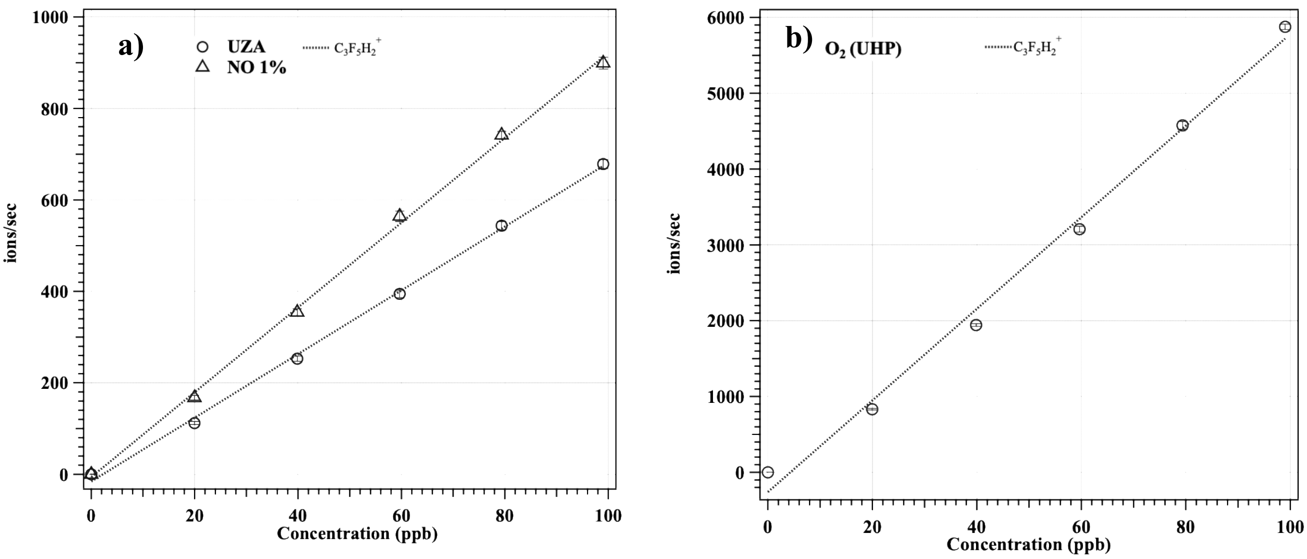

Figure S10. Calibration plot for PFP, where the signal intensities in ions/sec are plotted as a function of concentration (ppb). **a)** shows the result for instrument sensitivity with NO^+/O_2^+ as the reagent ion, the circle represents UZA, and the triangle represents NO 1% as a source of the reagent ion. **b)** shows the result for instrument sensitivity with O_2^+ reagent ion, O_2 (UHP) is used as source for the reagent ion. The linear regression lines were used to derive sensitivity. The error bars represent the standard deviation for ions/sec and fall within the markers for each data point.

S3. Calculation of calibration gas concentration and limit of detection

$$Concentration = \frac{Flow\ rate\ of\ the\ PFAS\ reference\ mixture\ (PFAS,\ sccm)}{Flow\ rate\ of\ the\ zero\ air\ gas\ (sccm) + PFAS\ (sccm)} \times 10\ ppm \quad \text{(S6)}$$

Where 10 ppm is a concentration of the PFAS reference mixture.

$$Limit\ of\ Detection\ (LOD)\ \left(\frac{ions}{s}\right) = 3\sigma \quad \text{(S7)}$$

Where, σ is the standard deviation of the background signal in ions/s.

$$\text{LOD (ppb)} = \frac{3\sigma}{Sensitivity} \quad \text{(S8)}$$

Where sensitivity is in the units of ions s^{-1} ppb^{-1}

Table S2. Intercomparisons of the sensitivity (ions s^{-1} ppb^{-1}) of selected PFAS using NO^+/O_2^+, O_2^+, and H_3O^+ reagent ions, respectively. The uncertainties of the sensitivities denote the standard errors from linear regression of the calibration curve for each PFAS.

			Sensitivity (ions s^{-1} ppb^{-1})							
**Compound**	**Charged ion**	**m/z**	**UZA**	**R^2**	**NO 1%**	**R^2**	**O_2**	**R^2**	**H_3O^+**	**R^2**
**HFO**	$C_3F_4H^+$	113.0009	520 ± 10	0.999	600 ± 20	0.994	3380 ± 30	0.999		
	$C_3F_4H_2^+$	114.0009	81.5 ± 1.7	0.998	122 ± 2	0.998	904 ± 15	0.999		
	$C_3F_4H_2^+$ plus $C_3F_4H^+$		600 ± 10	0.999	730 ± 30	0.995	4280 ± 40	0.999		
	$C_3F_3H_2^+$	95.0103							97.5 ± 0.6	0.999
**HFP**	$C_3F_6^+$	149.0087	330 ± 10	0.997	460 ± 10	0.998	3730 ± 60	0.999		
	$C_3F_5^+$	130.9914	1230 ± 10	0.999	1470 ± 50	0.995	6920 ± 80	0.999	278 ± 3	0.999
	$C_3F_6^+$ plus C_3F^+		1560 ± 10	0.999	1930 ± 60	0.996	10546 ± 131	0.999		
**PFB**	$C_4F_8^+$	199.9866	145 ± 6	0.994	215 ± 5	0.998	1570 ± 30	0.995		
	$C_4F_7^+$	180.9882	71.6 ± 0.9	0.999	125 ± 4	0.996	295 ± 3	0.999	1.2 ± 0.03	0.998
	$C_4F_8^+$ plus $C_4F_7^+$		215 ± 5	0.997	341 ± 6	0.999	1780 ± 60	0.996		
**PFPe**	$C_5F_{10}^+$	249.9834	2.7 ± 0.1	0.993	4.8 ± 0.10	0.998	44.2 ± 0.6	0.997		
	$C_5F_9^+$	230.9850	21.3 ± 0.6	0.997	73.7 ± 2.2	0.996	35.5 ± 0.3	0.999	1.6 ± 0.4	0.998
	$C_5F_{10}^+$ plus $C_5F_9^+$		23.9 ± 0.6	0.998	78.3 ± 2.2	0.997	77.9 ± 1.3	0.999		
**PFP**	$C_3F_5H_2^+$	133.0071	6.7 ± 0.1	0.998	9.3 ± 0.2	0.999	60.4 ± 2.5	0.993		

			Sensitivity (ions s^{-1} ppb^{-1})							
**Compound**	**Charged ion**	**m/z**	**UZA**	**R^2**	**NO 1%**	**R^2**	**O_2**	**R^2**	**H_3O^+**	**R^2**
**PMVE**	$C_3F_6O^+$	165.9847	15.5 ± 0.4	0.996	23.2 ± 0.6	0.999	179 ± 3	0.999		
	$C_3F_3O^+$	108.9895							240 ± 2	0.999
**PEVE**	$C_4F_8O^+$	215.9783	58.4 ± 2.0	0.996	84.6 ± 1.5		590 ± 10	0.999		
	$C_4F_5O^+$	158.9863							5.3 ± 0.1	0.998
**PPVE**	$C_5F_{10}O^+$	265.9783	23.7 ± 0.9	0.994	35.2 ± 0.7	0.999	250 ± 5	0.999		
	$C_5F_7O^+$	208.9831							3.4 ± 0.1	0.998

Table S3. Intercomparisons of the LOD (10 s) and LOQ of selected PFAS using the NO^+/O_2^+, O_2^+, and H_3O^+ reagent ions, respectively. The standard deviation is estimated from the variability in the sensitivity of each PFAS.

			LODs and LOQs (ppt)							
**Compound**	**Charged ion**	**m/z**	**UZA**		**NO 1%**		**O_2 (UHP)**		**H_3O^+**	
			**LOD**	**LOQ**	**LOD**	**LOQ**	**LOD**	**LOQ**	**LOD**	**LOQ**
**HFO**	$C_3F_4H^+$	113.0009	5.6 ± 0.1	18.5	5.5 ± 0.3	18.2	2.90 ± 0.02	9.4		
	$C_3F_4H_2^+$	114.0009	11.8 ± 0.3	39.0	7.2 ± 0.2	23.8	3.0 ± 0.03	10.0		
	$C_3F_4H_2^+$ plus $C_3F_4H^+$		5.4 ± 0.1	17.8	4.9 ± 0.2	16.2	2.5 ± 0.1	8.2		
	$C_3F_3H_2^+$	95.0103							84 ± 0.1	277.2
**HFP**	$C_3F_6^+$	149.0087	4.1 ± 0.1	13.5	3.8 ± 0.2	12.5	2.0 ± 0.02	6.4		
	$C_3F_5^+$	130.9914	8.3 ± 0.1	27.4	6.4 ± 0.3	21.0	2.20 ± 0.005	7.1	24 ± 0.3	78.5
	$C_3F_6^+$ plus $C_3F_5^+$		6.6 ± 0.1	21.8	5.1 ± 0.2	16.8	1.6 ± 0.1	5.3		
**PFB**	$C_4F_8^+$	199.9866	5.9 ± 0.2	19.5	3.9 ± 0.1	12.7	1.50 ± 0.03	4.9		
	$C_4F_7^+$	180.9882	20.3 ± 0.2	67.0	11.5 ± 0.4	37.8	7.3 ± 0.02	24.0	119.0 ± 3.0	357.0
	$C_4F_8^+$ plus $C_4F_7^+$		6.6 ± 0.10	21.8	5.1 ± 0.15	16.8	1.8 ± 0.03	5.9		
**PFPe**	$C_5F_{10}^+$	249.9834	55.4 ± 2.0	182.8	70.0 ± 1.5	231.0	9.5 ± 0.2	31.3		
	$C_5F_9^+$	230.9850	39.5 ± 1.2	130.3	13.4 ± 0.4	44.5	41.0 ± 0.4	134.5	513 ± 13	1693.0

			LODs and LOQs (ppt)							
**Compound**	**Charged ion**	**m/z**	**UZA**		**NO 1%**		**O_2 (UHP)**		**H_3O^+**	
			**LOD**	**LOQ**	**LOD**	**LOQ**	**LOD**	**LOQ**	**LOD**	**LOQ**
	$C_5F_{10}^+$ plus $C_5F_9^+$		35.3 ± 0.9	116.5	13.2± 0.5	44.6	19.0± 0.1	62.7		
**PFP**	$C_3F_5H_2^+$	133.0071	89.2 ± 1.3	294.3	102.0 ± 2.0	337.0	18.0 ± 1.0	59.4		
**PMVE**	$C_3F_6O^+$	165.9847	25.9 ± 0.6	85.8	23.2 ± 0.6	76.6	6.8 ± 0.1	22.4		
	$C_3F_3O^+$	108.9895							15.0 ± 0.3	49.5
**PEVE**	$C_4F_8O^+$	215.9783	6.0 ± 0.2	19.8	6.3 ± 0.2	20.8	2.80 ± 0.02	9.3		
	$C_3F_5O^+$	158.9863							228.0 ± 4.0	752.4
**PPVE**	$C_5F_{10}O^+$	265.9783	10.0 ± 0.4	33.0	9.5 ± 0.2	31.4	4.8 ± 0.04	15.8		
	$C_5F_7O^+$	208.9831							2840.0 ± 80.0	9372.0

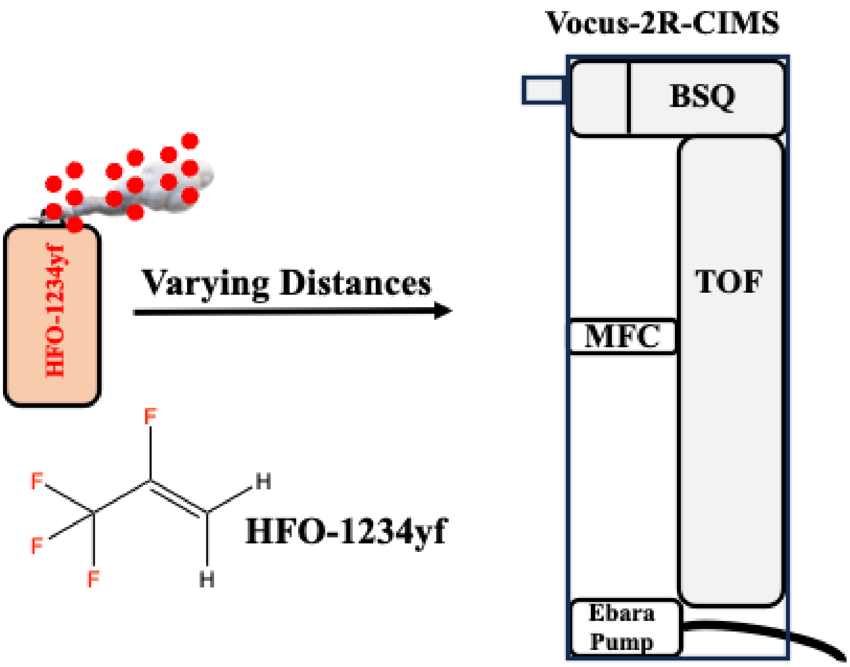

Figure S11. Schematic representation for the real-time measurement of HFO-1234yf. The puffs were generated at varying distances of 0.9, 1.5, 2.7, 5.2, 5.8, and 6.4 m from the Vocus-CIMS inlet.

S4. Determination of Air Exchange Rate

The air exchange rate for the room, expressed in air changes per hour (ACH), was provided by the building facilities team. It was calculated based on the measured supply and return airflow rate of 32.6 cubic meters per minute and the known laboratory volume of 258 m^3. Using these values, the air exchange rate was determined to be 7.6 h^{-1}
